# Adverse childhood experiences, family relationship and generalized anxiety in the youth population in Hong Kong

**DOI:** 10.1192/j.eurpsy.2021.984

**Published:** 2021-08-13

**Authors:** W.C. Tang, C.S. Wong, W. Chang, C.L. Hui, S.K. Chan, E.H. Lee

**Affiliations:** 1 Department Of Psychiatry, The University of Hong Kong, HK, Hong Kong PRC; 2 Department Of Psychiatry, The University of Hong Kong, Hong Kong, Hong Kong PRC

**Keywords:** youth population, adverse childhood experiences, family relationship, generalized anxiety

## Abstract

**Introduction:**

Adverse childhood experiences (ACEs) are shown to be risk factors for developing anxiety later in life. However, one’s family relationship acts as a protective factor between ACEs and anxiety.

**Objectives:**

The present study examines the interaction between ACEs and family relationship and their effect on generalized anxiety (GA) amongst the youth population in Hong Kong.

**Methods:**

Participants aged 15-24 were recruited from a population-based epidemiological study in Hong Kong. GA in the past two weeks was assessed using GAD-7, while ACEs were measured using the childhood section of Composite International Diagnostic Interview screening scales (CIDI-SC), encompassing parental psychopathology, physical, emotional, sexual abuse, and neglect before age 17. Family relationship was measured by the Brief Family Relationship Scale (BFRS). Linear regression and a two-way ANCOVA were conducted to examine the association between ACEs, family relationship and GA, while adjusted for age and gender.

**Results:**

633 (70.7%) out of 895 participants had any ACEs. ACEs significantly predicted GAD-7 scores (Β=1.272, t(891)=4.115, p<.001). Two-way ANCOVA reported a significant interaction effect of ACEs and family relationship on GA (F(1, 889)=4.398, p=.036), namely those who had any ACEs and poorer family relationship scored higher in GAD-7 (p<.001), whereas there was no difference in family relationship for those without ACEs on GA (p=.501).

**Conclusions:**

ACEs increases the vulnerability to GA later in life. However, its effect on anxiety decreases when one has a better family relationship. This suggests a possible moderating role of family relationship in developing GA among younger people.

